# Supplementary material for: Are carbon emissions trading and green financial instruments synergistic? -Comprehensive quantitative research based on content analysis
Source: PLoS One. 2024 Mar 7;19(3):e0298601. doi: 10.1371/journal.pone.0298601 (PMC10919601; doi:10.1371/journal.pone.0298601)
Supplement: S1 Data — (ZIP) [file pone.0298601.s001.zip › Data/地级市Z_F工作报告.zip!/╝¬┴╓/╝¬┴╓╩╨/└┤╫╘╣1⁄2╓┌║┼í╛┬φ┐╦╩2╛▌═°í┐_2012.docx]

各位代表：

　　现在，我代表市政府向大会报告工作，请予审议，并请政协委员提出意见。

　　一、2011年工作回顾

　　去年是“十二五”开局之年。我们在省委省政府和市委的正确领导下，在市人大、市政协的大力支持下，按照科学发展、富民强市的总要求，坚持“三化”统筹，实施“三动”战略，全面开展“五项攻坚”、奋战三季度和决战四季度等系列活动，圆满完成了市十四届人大四次会议确定的各项任务，是我市重点工作成效突出、发展成绩尤为显著的一年。预计全市地区生产总值达到2278亿元，同比增长26.5%；全口径财政收入245.1亿元，增长27.5%；地方级财政收入101.9亿元，增长39.3%；城镇居民人均可支配收入19300元，农村居民人均纯收入7649元，分别增长14%和16%。

　　---产业结构调整步伐加大。三次产业比重调整为9.6：50.4：40。汽车、冶金、农产品加工等支柱优势产业升级改造进程加快，以碳纤维为主的新材料等战略性新兴产业科技创新和发展能力较大增强。农业和农村经济发展态势良好。粮食产量迈上100亿斤历史新台阶；畜牧、园艺特产、林业产值分别增长16%、20%和20%。长白山食品等20个重点产业化项目竣工投产，市级以上龙头企业达到185户。实施农田水利建设658项，灾后恢复重建任务基本完成。县域生产总值、地方级财政收入分别增长27.6%和45.9%。现代服务业发展水平较快提升。实现社会消费品零售总额841.3亿元，增长23%。财富广场等9个大型商贸项目投入运营，限额以上企业发展到789户；外贸进出口总额增长

45.4%。内陆港联检大楼建成使用。光大银行分行正式运营，永大集团成功上市。特色旅游精品线路及生态游、民俗游等新产品积极拓展，雾凇冰雪节、首届松花江彩灯文化节等大型节庆活动对外影响力明显扩大，旅游总收入增长30%。

---社会事业实现新发展。实施市级以上重大科技成果转化和重点科技攻关项目180项，国家碳纤维产业基地建设取得较大进展，荣获全国科技进步先进市称号。完成75所中小学校舍重建改造，素质教育、职业教育保持全省先进水平，高等教育健康发展。基层医疗卫生机构综合改革全面完成，基本药物零加价实施范围扩大，重大传染病防控和卫生监管有效加强，医疗保障能力和服务水平得到有力提升。市歌舞团等文化品牌效应增强，“北山揽胜”彩灯组创吉尼斯世界纪录，农家书屋覆盖所有行政村，高质量完成第十二届全国冬运会雪上赛事筹备工作，纪念建党90周年等各类文体活动持续开展，广泛营造了健康、和谐、向上的社会氛围。

　　---民主法制建设进一步加强。主动接受人大、政协监督，定期向人大报告、政协通报工作；认真办理人大代表建议、政协提案，并积极纳入政府工作思路和决策之中。“六五普法”规划启动实施，制定地方性法规和政府规章15部，科学民主决策机制日趋完善，法治政府建设进程继续加快。深化政府信息公开，健全“一站式”服务平台，行政审批权相对集中改革成果不断扩大。强化监管服务机制，软环境得到新的优化。“三帮双促”和“我能为企业做些什么、能给群众带来些什么”等活动深入开展，政府的服务理念、工作效能和整体形象发生了积极变化。昌邑区社会管理创新试点经验在全市推广。强化消防、生产、交通、食品药品等专项排查整治，安全生产防控和应急救援体系有效健全。落实领导包案和信访接待日制度，731件信访积案得到妥善解决。严厉打击各种犯罪活动，社会安全感进一步提升。双拥共建活动深入开展，军政军民团结更加巩固。

　　过去一年，我们之所以从年初全省靠后排位迈入先进行列，主要是通过组织实施“五项攻坚”和“三帮双促”活动，用重点工作的率先突破强力带动了全局跃升。

　　(一)全力实施项目建设攻坚，固定资产投资拉动作用明显加大

　　新建续建投资3000万元以上重点项目876项，完成全社会固定资产投资1553亿元，增长31.5%。重点项目大力推进，32万吨苯乙烯、中钢2000吨碳纤维、华润雪花啤酒异地迁建等220项纳入责任推进体系的重大工程快速建设，建龙精品钢、吉林碳谷5000吨碳纤维原丝等170个工业项目建成投产。投资结构进一步优化，社会民生、城市基础设施、能源交通领域项目成效明显；现代服务业项目比重提升；战略性新兴产业、产业链延伸项目进一步增多，工业项目投资保持全部投资的60%以上。项目布局更加合理，新开工项目更多集中在各类开发区、重要功能区和重点节点镇，产业园区化、集聚化特征日趋鲜明。

　　（二）强力实施招商引资攻坚，“外引”、“上争”成果显著扩大

　　创新招商方式，强化责任推进机制，定点招商、产业招商、专业招商及第七届东博会、京沪浙、东南亚、德法荷等一系列境内外招商活动取得重大成效，招商引资到位资金590亿元。美国亨斯迈、德国赢创、日本住友、马来西亚完美等世界500强和跨国公司，恒天、中粮、万科等国内知名企业落户我市，松花湖国际旅游度假区、中油数据中心、生物质能源等一批规模大、质量好、拉动力强的重大项目签约落地，全年新开工3000万元以上招商项目368项；实际利用外资4.9亿美元。加大“上争”力度，成为全国节能减排财政政策综合示范城市和国家电子商务示范城市，争取国家和省各类财政资金109.5亿元。

　　（三）大力实施提速增效攻坚，工业经济发展水平持续提高

　　努力克服生产成本加大等不利因素影响，强化服务和政策扶持，工业发展速度加快、效益提高，整体实力得到新的增强。完成规模工业总产值2800亿元、增加值810亿元，分别增长38%和33%；工业贷款增长29.2%，高于全市平均水平17.1个百分点。昊融有色金属、康乃尔化工等骨干企业产出能力较大提升，百户重点企业完成产值1510亿元。中小企业成长步伐加快，实现民营经济主营业务收入3300亿元，实缴税金87亿元，分别增长35%和42%。大黑山钼矿扩产等130个建成项目达产达效率达到87.7%。超额完成省下达的节能减排任务。

　　（四）努力实施城市建设与管理攻坚，城镇化发展进程有效加快

　　完成城建投资260亿元；房地产业和建筑业增加值分别增长34.7%和43.7%，创历史最高水平。坚持规划先行，长吉一体化、哈达湾区域搬迁改造等重大规划编制完成，“一核、两线、十大功能区”的城市发展战略思路进一步明晰，可持续发展的产业布局和现代化大城市框架有效构建。开工江城大桥、雾凇大桥及高架桥等城建重点工程136项，解放西路、温德桥续建等74项竣工使用。吉沈高速公路通车，建成各级各类公路721公里。黄旗街等4条供水管线、滨江东路等5条污水管线完工，截流污水吐口7处；铺设天然气网线54公里。房地产业健康发展，开发面积852万平方米，竣工609万平方米。完成清水绿带恢复重建，新增绿地72.3公顷，城市空气质量、水质得到新的改善。深入开展城市管理创优年活动，占道经营、渣土管理等综合整治成效明显，城市的整体形象和市容市貌得到进一步改观。县级城市的发展水平和公共服务能力快速提升，孤店子等5个“三化”统筹试点镇建设速度加快，新农村建设走在全省前列。

　　（五）着力实施民生改善与提高攻坚，民生事业取得较大成绩

　　在年初落实十大民生任务的基础上，将民生和社会事业项目拓展到100项，总投入达到135.5亿元，是近年来民生投入最大、范围最广、群众得实惠最多的一年。就业工作有效加强，开发城镇就业岗位13.6万个，新增就业9.3万人，劳务输出64万人次。社会保障体系进一步完善，“五七家属工”参加养老保险工作基本完成，在全省率先实现城乡养老保险制度全覆盖；将7.6万名并轨和改制国企退休人员免费纳入基本医疗保险，城镇居民医保报销比例和支付限额提高。健全社会救助机制，城市低保标准提高到每人每月300元，农村低保标准提高到每人每年1650元；城乡低保补助与物价水平联动机制形成；社会救助“一站式”服务全面实现。困难劳模补助制度建立。养老援助计划稳步实施。棚户区和危旧楼改造建设回迁房屋144万平方米，1.8万户居民住房得到根本改善；建设廉租住房和公租房27万平方米，为3.7万户居民发放廉租住房租赁补贴；完成6485户农村居民泥草房改造。暖房子工程改造热网415公里，并网827万平方米，集中供热率达到80.6%；完成居住建筑节能改造593万平方米，8.3万户、24.3万城镇居民受益。出行环境得到优化，维修改造滨江北路等32项路桥工程，更新公交车100台。菜篮子工程新建温室大棚4200栋，新增蔬菜生产能力1亿斤。推行农户科学储粮仓2.5万套，建设农村户用沼气池4500座，又解决19.6万农村人口安全饮水问题。同时，“三帮双促”活动解决各类问题近2万件，扎扎实实地取得了知民情、解民忧、暖民心的良好社会效应。

　　各位代表，在复杂的外部环境下取得这些成绩，是省委省政府和市委正确领导的结果，是市人大、市政协大力支持的结果，是全市上下共同努力的结果。广大干部群众以高度的热情积极投身到各项工作中，戮力同心，开拓进取，充分凝聚了加快发展的整体合力，有效营造了埋头苦干抓落实、奋力争先促发展的社会氛围。在此，我代表市人民政府，向全市各族人民，向辛勤工作在各条战线的广大建设者，向各级人大代表、政协委员、各民主党派、工商联和社会各界人士，向中省直单位、驻吉部队指战员和武警官兵，以及始终关心支持吉林市发展的海内外朋友，表示诚挚的感谢和崇高的敬意！

　　回顾过去的一年，我们也清醒地认识到，发展中既面临着总量不大、结构不优、综合实力不强的根本性矛盾制约，也存在着一些急需破解的现实问题：中小企业融资难比较突出，部分骨干企业对经济的持续贡献能力亟待提升，开发区引领带动作用需要进一步发挥，改善民生还需加大工作力度。对于这些矛盾和问题，我们一定高度重视，认真加以解决。

　　二、2012年工作安排

　　今后一个时期，是我市加快转变发展方式、全面建设小康社会的关键时期。市十二次党代会在深刻把握宏观环境变化的基础上，对未来五年发展做出全面部署，提出了实现经济总量、产业结构、民生改善、城市形象、文化发展五大新跨越的宏伟目标。完成这一目标，今年是极为关键的一年。我们一定按照科学发展、富民强市的总要求，牢牢抓住发展和民生两个关键，统筹“三化”建设，落实“三动”战略，突出重点，突破难点，以“发展上水平、干部下基层”为主旋律，以十大功能区建设和县域经济发展为核心，全力实施“十项攻坚战”，切实开拓经济社会又好又快发展的新局面，努力让城乡广大居民生活得更加美好。

　　主要预期目标：地区生产总值达到2690亿元，同比增长18%；规模工业增加值970亿元，增长20%；社会消费品零售总额1010亿元，增长20%；全口径财政收入增长20%；地方级财政收入增长25%；城镇居民人均可支配收入和农村居民人均纯收入增长16%。节能减排完成省下达任务。

　　（一）突出空间布局优化，全面推进十大功能区领先突破

　　规划建设十大功能区，是市委市政府为优化生产力布局、拓展发展空间、完备城市功能、促进产业集聚升级做出的重大战略决策。作为未来发展的产业核心区域和支撑所在，要举全市之力加快开发建设，带动经济的快速发展和城市功能的不断提升。

　　科学编制完善相关规划。高标准完成高新北区、金珠工业区、南部新城、松花湖旅游区等功能区专项规划和相关区域控制性规划编制，启动经济开发区新一轮规划修编，科学明晰各功能区基本定位和发展目标。中心城市建成区面积力争扩大10平方公里以上。

　　统筹推进基础设施建设。按照总体规划、适度超前、创新方式、多元投资、分步开发的原则，加大土地征收整理力度，实施供水、供电、供热、供气、道路等80项基础设施工程，完成投资60亿元以上。

　　有效加速产业集聚步伐。实施产业项目160项，总投资1100亿元以上，其中，10亿元以上项目25项，亿元以上项目106项。高新南区，围绕建设百万辆整车生产基地，组织实施一汽吉林40万辆整车、新湖通田50万辆整车及零部件配套等一批重点项目。高新北区，立足打造战略性新兴产业聚集区，积极探索实行市场化运作、公司化管理模式，启动30平方公里起步区建设，开工万丰产业园、长久改装车等一批产业项目。经济开发区，以精细化工、新材料、生物三大产业为重点，推进碳纤维产业化体系建设，加大12万吨环氧乙烷、1.5万吨聚酰亚胺树脂等项目实施力度。化工循环经济示范园区，按照产业发展链式化要求，深化与亨斯迈、赢创、林德等跨国公司的合作，加速形成产业集群发展新优势。金珠工业区，积极承接哈达湾区域产业转移，开工100万吨冷轧钢、50万吨大口径焊管等一批重点项目。哈达湾服务业集中区，有序推动冀东水泥搬迁和棚户区征收，着手启动晨鸣纸业搬迁工作。南部新城，加强基础设施建设，配套完善商贸、旅游、文化、教育、医疗等功能，快速打造以居住休闲、高端服务业为主的新城区。松花湖旅游区，有效整合区域旅游资源，规划建设万科松花湖国际旅游度假区、朱雀山开发等重点项目。北大壶体育旅游开发区，以承办第十二届全国冬运会雪上赛事为契机，强力打造北大壶品牌，建设国际一流的赛事基地和休闲旅游度假区。中新吉林食品区，以推进中新合作进程为核心，加快起步区建设步伐，规范管理无规定疫病区，积极引进新加坡淡马锡、吉宝和中信、中粮等战略合作伙伴，实施珍宝中成药保健品等农产品加工项目，为构建国际一流的安全健康食品生产示范区、现代农业发展示范区、“三化”统筹示范区和长吉图开发开放战略先行先试示范区

奠定基础。

　　（二）深入实施“三动”战略，全面增强科学发展的动力支撑

　　切实加大固定资产投资和项目建设力度。大力开展“项目建设年”活动，开工投资3000万元以上重点项目900项，其中亿元以上项目420项；建成投产350项。完成全社会固定资产投资2000亿元以上，增长30%。以滚动实施50项投资10亿元以上重大产业工程和200项投资亿元以上重点项目为主要支撑，全面加快各领域项目建设步伐，在保持工业投资比重的同时，将服务业投资比重提升到30%以上。围绕工业强市建设，组织实施丙烯产业链、整车制造及零部件、碳纤维系列产品等一批工业重点项目；围绕现代服务业跃升发展，加快建设商业综合体、星级酒店、数据处理中心等一批服务业重点项目；围绕城乡承载能力有效增强，着力抓好城市道路桥梁、市政工程等一批基础设施重点项目；围绕社会民生事业持续发展，快速推进教育、医疗、文化等一批社会民生重点项目。

　　大力抓好招商引资工作。新开工投资3000万元以上重点招商项目超过350项；招商引资到位资金770亿元，增长30%。一是加强招商项目谋划。准确把握市场形势和产业、资源特色，包装和推介一批重大的拟招商项目，年内储备亿元以上项目200个。二是强化园区和产业招商优势。发挥十大功能区招商主力军作用，突出主导产业和骨干企业，下力气引进延伸产业链条、扩大配套加工项目，推动招商引资向招商“选”资的有效转变。三是明确主攻方向。瞄准具有产业转移趋势的发达国家和先进地区，着力引进一批有实力、有信誉的战略投资者，在落地项目数量和质量上取得重大突破。

　　努力加快创新步伐。强化政策引导，加大投入力度，健全以企业为主体、市场为导向、产学研相结合的科技创新体系。鼓励技术创新和专利申请，组织实施高性能纤维、镍氢动力电池等一批重大科技成果转化和重点科技攻关项目；深化与省科技厅工作会商制度，积极推进东北电力大学、北华大学、化工学院等高校建设科技园；强化与吉林大学、中科院等高校和科研院所的产学研合作，培育引进一批金融、经营管理等高层次人才、紧缺人才和适用性人才。充分利用长吉一体化先行先试的契机，按照国际化、市场化、企业化建设模式，有效探索功能区实施公司化经营的管理体制；努力创新投融资、人才引进、建管市场化运作、收入合理分配等新机制，为经济社会发展提供有力的体制机制保障。

　　积极争取政策性资源。拓宽各领域信息渠道，准确把握政策资金取向，强化对上衔接沟通，更大程度获得国家和省的支持。围绕建设节能减排财政政策综合示范城市，着力做好方案报批和项目谋划、包装、申报等前期工作，组织实施公共机构节能、化工园区循环化改造、松花湖生态整治等一批重点示范工程。抓住国家编制老工业基地调整改造规划的机遇，争取把哈达湾老工业区列入优先启动示范区，并努力将我市重点产业、重大项目等更多内容纳入国家的整体规划。

　　（三）推进产业协调发展，全面提高经济增长质量效益

　　着力增强工业竞争实力。以壮大骨干企业和培育中小企业为重点，做大做强支柱优势产业，大力发展战略性新兴产业，加速工业经济的转型升级进程。一是实施百户重点企业跃升计划。通过上新项目、技改扩能等途径，进一步提升百户企业市场竞争力和综合发展实力，销售收入50亿元以上、5-50亿元企业分别发展到5户和35户；百户重点企业实现产值1740亿元。二是实施中小企业成长壮大工程。加大政策支持力度，强化创业服务体系和综合服务平台建设，全面营造中小企业发展的服务环境；完善治理结构，拓宽多元融资渠道，有效提升中小企业管理和资金瓶颈化解能力；依托骨干企业和重大项目，引导中小企业发展精深加工和协作配套，推进民营经济新一轮腾飞。新增规模工业企业100户以上，纳税超千万元企业增加到60户。三是实施战略性新兴产业发展计划。搭建碳纤维公共技术创新平台，加强碳纤维下游制品研发，加快国家碳纤维产业基地建设；突出抓好100项投资3000万元以上战略性新兴产业项目，组织推进50种新产品规模化生产，战略性新兴产业比重比去年提高1.5个百分点。四是抓好建成项目达产达效。推进去年以来建成投产的重点项目达产达效，达产达效率力争达到80%。五是深入开展企业优质服务年活动。健全工业生产调度和协调服务平台，积极创新产需衔接、银企对接、要素保障等服务举措，有力保证企业生产经营的顺利进行。

　　加大县域经济突破步伐。五县（市）生产总值、地方级财政收入分别增长20%和25%以上。一是推进县域工业化。加快经济结构战略性调整，积极探索发展新兴替代产业，促进舒兰、磐石、蛟河、桦甸资源型城市有效转型；突出县域工业集中区发展，强化规划引导，加大基础设施建设，推动更多项目入区发展；依托产业资源特色，建设凯迪生物质汽柴油等一批县域工业重点项目，加速形成县域特色化、集群式的产业发展优势。二是推进县域城镇化。发挥5座县（市）城的区域次级中心功能，加强城市建设管理，协调发展服务业和社会事业，切实增强综合承载功能和辐射吸纳能力。着力抓好节点城镇发展，优先建设长吉南北轴线节点城镇，提升岔路河等5个试点镇建设水平，有序推动夹皮沟、烟筒山、天岗、吉舒等一批区位较好、产业带动功能较强的特色重点镇、中心镇发展，快速构建梯次分明的现代城镇体系；深入实施“千村示范、万村提升”工程，着力抓好“三化”统筹示范村建设，壮大村级集体经济，进一步加快新农村建设进程。三是推进农业现代化。建设63个粮油高产示范基地，粮食产量保持100亿斤阶段水平；新建扩建200个标准化养殖场，壮大蛟河黑木耳等特产业园区规模；发展生态林业，推进实施第二个十年绿化美化吉林大地规划；建设一批重点农业产业化项目，年销售收入亿元以上农产品加工企业发展到30户。加强农业科技创新与新技术推广，提升农业机械化、信息化水平，组织实施病险水库除险加固、中小河流治理、大中型灌区节水改造等水利基础设施416项，切实增强农业综合生产能力。深化乡村债务化解、土地流转等各项改革，进一步激发县域经济发展的活力。

　　加快服务业跃升发展。修订完善服务业发展规划，出台落实扶持政策，进一步提高服务业对经济发展的贡献度。一是大力发展文化旅游产业。用文化提升旅游品质、旅游彰显文化内涵，推进文化与旅游产业融合发展。深度挖掘自然、民俗、历史、人文等文化内涵，打造四季各具特色的文化旅游产品，形成更加完整的文化旅游产业链条；重点开发以历史文化为主题的龙潭山、以自然生态文化为主题的朱雀山、以休闲娱乐文化为主题的松花湖、百里清水绿带等一批精品景区；加快旅游配套设施建设，提高旅游服务接待能力；高水平举办好雾凇冰雪节等大型节庆活动，强化宣传推介力度，深度拓展境内外客源市场，促进文化旅游资源优势向经济优势的快速转化。实现文化旅游总收入273亿元。二是加快发展商贸流通业。科学规划商业网点，优化商业布局，抓好欧亚城市商业综合体等一批重点项目建设，着力打造各具特色的区域商贸中心。积极引进国内外大型商贸企业，大力发展新型、高端商贸业态，培育销售额10亿元以上企业3户、5亿元以上5户、亿元以上15户，限额以上企业力争突破1000户。支持外贸企业发展，加快化工、电子等外贸出口基地建设。三是快速推进现代物流业。完善内陆港、统泰、越北等重点物流中心功能，规划建设城区西南部大型综合物流园区，壮大一批物流骨干企业，构建科学完备的现代物流网络。四是积极发展金融业。引进2户以上外埠银行来吉开办业务，新发展3户小额贷款公司和2户担保公司；推进昊宇电气、英联生物、科龙建筑等企业尽早上市；加快农村商业银行建设步伐；深化政府投融资体系改革，引进股权投资基金，支持都邦公司实施股权改革，切实提高金融对经济发展的支撑能力。

　　（四）加强城市建设管理，全面提升城市综合承载功能和整体形象

　　大力抓好城市基础设施建设。规划实施城建重点项目180项，完成城建投资249亿元。加强规划编制工作，做好控制性详细规划，有效发挥规划的导控功能。完备道路交通体系，启动长吉北线改造、秀水大桥、沙河子立交桥，续建吉图珲客运专线、绕城高速公路、金珠大桥、吉钢大路，完成江城大桥、雾凇大桥、中东南路、苏合街等工程建设。加快功能性城市设施建设，实施人民剧院、人民医院二期、中小学艺术活动中心等重点项目；新建3条污水管线，启动污水处理厂二期扩建；推进房地产健康持续发展，开发810万平方米，竣工550万平方米。

　　全面开展“城市管理提升年”活动。规范城市资源开发利用，严格基本建设审批程序和监管，保障建设项目依法合规。加强生态环境建设，移植大树7000株进城，栽植花卉200万株，新增绿地40公顷；对越山路、解放大路、吉林大街中南段等重点街路建筑物装饰美化；高品位提升一江两岸、临江大桥等主要景观路和桥体的亮化水准，完成龙潭山等山体的美化亮化；强化城区卫生保洁；加大公用设施管护力度。科学调整交通流量、整顿交通秩序、深挖管理潜力，下大力气改善主街路、重要节点的交通状况。积极构筑数字化、精细化、动态化城市管理网络系统，努力推进市容管理常态化、标准化；针对违章建筑、广告牌匾等重点问题，全面开展市容综合整治行动；完善松江路等5条景观路，改造辽北路等9条街路为标准化街路，明显提升城市的整体形象，进一步优化人民群众的生产生活环境。

　　（五）加大改善民生力度，全面扩大民生事业发展成果

　　进一步增大民生投入，组织实施十个方面130项改善民生事项。

　　第一，就业增收方面。开发城镇就业岗位11.5万个，新增就业9万人，其中有针对性开发1万个适应性就业岗位，安置万名困难家庭成员就业。培训农村劳动力、城镇失业人员2.6万人。发放小额担保贷款1.2亿元，扶持创业者2100人；帮扶千户有创业愿望、具备创业能力的困难家庭创业。将没有参加其他各类养老保险的60周岁以上城乡居民和城乡低保对象、丧失劳动能力的重度残疾人、城镇困难居民家庭成员，免费纳入新型农村社会养老保险和城镇居民社会养老保险。

　　第二，安居保障方面。加大棚户区和危旧楼改造力度，建设回迁住房1.39万套、95.9万平方米；建设廉租房和公租房24.6万平方米，为3.4万户居民发放廉租住房租赁补贴。继续实施“暖房子”工程，建造热网295公里，完成居住建筑节能改造1142万平方米，使16.3万户家庭受益。建设燃气管网10公里，发展天然气用户1.2万户。

　　第三，医疗惠民方面。在全市基层医疗卫生机构实施510种基本药物零加价。城镇居民医疗保险、新农合年度报销最高额度分别提高到5.5万元和8万元；新增单病种结算病种10个以上。建设远程医学中心，降低群众域外就医成本，推动全市医疗技术水平进一步提升。组织两次以上支援农村、走进社区大型义诊活动，免费为城乡群众送医送药。

　　第四，困难救助方面。城市低保标准提高到每人每月330元以上，农村低保标准提高到每人每年1800元以上。提升医疗救助标准和“一站式”服务水平，将低保对象、城镇困难居民家庭患者在规定病种封顶线内的自付医疗费用比例降低到25%以内。低保家庭成员就医，享受市直三甲医院基本药物零加价、大型设备检查费减免50%和市直属医疗机构住院床位费减免50%的政策。深入开展慈善救助活动，及时救助特殊困难群众1万人次和贫困患者1千人次以上。

　　第五，教育助学方面。对低保、城镇困难居民家庭学生给予一定资助：普通高中品学兼优学生每人每年资助1500元，中等职业学校一二年级学生每人每年免除学费2000元，新入学大学生分类资助5000元或3000元，初中毕业女学生免费提供职业技术教育培训。对低保家庭中小学生免费提供在校午餐。

　　第六，文体生活方面。开展公益免费电影放映活动，进社区、广场放映1000场，进乡镇村屯放映4200场。免费公演经典童话剧50场。新建16处标准化体育健身场地，为10个标准化社区和20个行政村安装健身器材。增设“江城百姓”讲坛等民生专题栏目，反映市民心声，服务百姓生活。

　　第七，便民畅通方面。维修改造延安路等10条主街路和100条小街小巷；农村水泥路“村村通”率达到96%以上。完善小街小巷和居民小区路灯设置。优化整合市区25条公交线路，延长公交线路和营运时间，再更新100台公交车辆，方便市民出行。

　　第八，环境整治方面。开展春秋季城市环境卫生综合整治，在60条街路实施水冲洗和洒水降尘作业。以整治流动摊点、交通秩序等为重点，大力净化校园周边环境。环境空气质量好于二级标准天数超过320天，城市饮用水水质达标率达到100%。新建农村户用沼气池4000座。

　　第九，平安创建方面。健全制度机制，实施综合治理，切实保障接送学生车辆安全。加大食品质量监管力度和监测频次，及时向社会发布检测结果，确保群众吃上放心食品。新建农村饮水安全工程266处，再解决16万农村人口安全饮水问题。

　　第十，公共服务方面。继续实施“菜篮子”工程，新建2000栋温室大棚，新增蔬菜生产能力5000万斤；建设市粮食批发市场交易大厅和35个平价副食品商店，发挥平抑市场价格、保障居民正常生活供应的功能。新建社区居家养老服务站57个，实现城区社区居家养老服务站全覆盖。建立居民诉求绿色通道，快速办理群众反映的民生问题。

　　（六）深化改革进程，全面推进社会事业协调发展

　　努力推动文化大发展大繁荣。以高度的文化自觉和文化自信，坚持文化事业和文化产业两手抓，深挖文化内涵，展示文化亮点，提升文化品位，最大限度地满足人民群众日益增长的精神文化需求。建设全民健身中心、传媒中心等一批重点文化设施，做好历史文化遗址保护和本体展示工作。加强县（市）图书馆、文化馆和博物馆建设，推进县（市）级公共文化设施逐步免费开放。创新形式、扩大规模，高标准举办好第二届松花江彩灯文化节；以松花江之夏广场文化周、国际冬泳邀请赛等品牌活动为引领，广泛开展好各类群众性文化活动和全民健身活动。突出山水文化、地域文化和现代文化，做大做强演艺娱乐、出版发行等传统文化产业，扶持文化创意、动漫游戏等新兴文化产业，培育壮大一批在全省有分量、在全国有影响的骨干文化企业，推动文化产业向特色化、品牌化、规模化发展。加强精神文明建设，进一步提升城市文明程度和市民文明素质。

　　进一步提高城乡教育发展水平。整合教育资源，

加强学校标准化建设，促进义务教育均衡发展；突出学生德育和实践教育活动，狠抓教师队伍建设和教学质量管理，不断深化素质教育发展进程；结合产业发展趋势和功能区建设，推进职业教育市场化改革，打造职教集团和职教园区；扩大学前教育资源，积极发展学前教育；加强工作扶持，提升高等学校办学质量。

　　加快发展医疗卫生事业。推进医药卫生体制改革，巩固基层医疗卫生机构综合改革成果，抓好以县级医院为重点的公立医院改革。完善城乡医疗保障体系，提升区域医疗中心和社区卫生服务中心服务质量，加强农村三级网络规范化建设；认真做好各种传染病防控，强化医疗药品市场监管，进一步完善公共卫生服务体系。保持人口和计划生育工作全省先进水平。

　　（七）强化社会管理创新，全面开拓和谐社会发展新局面

　　加强基层社会管理。推行社区网格化管理，建立社区社情民意调查、公开服务承诺、反馈督办等制度，加强社区工作队伍建设，完善社区运行管理机制。突出流动人口服务管理、弱势群体帮扶、“两新”组织建设等重点方面，抓好社区管理任务落实。开展领导干部定期深入社区活动，积极帮助社区解决用房不足等实际问题。组织开展100个行政村社区化管理试点，不断扩大农村基层组织建设的工作成效。

　　扎实抓好安全生产。严格落实各级各类安全生产目标责任和奖惩机制，强化长效监管和应急救援体系建设。高度重视生产安全、消防安全、交通安全和食品药品安全，加大集中检查和专项整治力度，及时消除各种安全隐患，不断提高保障公共安全和处置突发事件的能力水平。

　　认真做好社会稳定工作。健全矛盾纠纷排查调处机制，完善领导干部联系群众和信访接待日制度，有计划、有步骤地化解企业改制、土地征收等重点信访问题。强化社会治安综合治理，提升公安信息化、科技化水平，严厉打击各种刑事犯罪、经济犯罪和“法轮功”等邪教组织；加大治安防控体系建设力度，全面开展社会管理大巡防工作，进一步提升人民群众的安全感和社会满意度。

　　支持国防和军队建设。加强国防教育，增强全民国防意识。提高民兵和预备役部队建设水平，支持驻吉解放军和武警、消防、森警部队建设。深入开展双拥共建活动，认真落实优抚安置政策，努力解决驻吉部队军官家属就业、子女入学等实际困难，巩固军政军民团结，不断拓宽军民融合式发展领域。

　　三、切实加强政府自身建设

　　新的形势、新的任务，对政府工作提出了新的更高要求。我们一定强化政府自身建设，牢记使命，依法行政，勤勉尽责，努力建设为民、务实、高效、清廉的人民满意政府。

　　着力加强民主法制建设。把依法行政贯穿于政府工作的全过程，严格按照法定权限和程序履行职责。有效健全科学民主决策机制，凡事关全局重大项目和民生重要事项，都坚决执行公众参与、专家论证、合法性审查和集体决策制度，使政府决策更加科学、经得起历史检验。加大“六五”普法力度，增强全社会的法律意识；提高政府立法质量，加强和改进政府制度建设。理顺行政执法机制，规范行政执法行为，严格执法监督和责任追究。自觉接受人大及其常委会法律监督、工作监督和政协的民主监督，高度重视司法监督、舆论监督和社会公众监督，加强政府内部层级监督，使政府各项工作真正在依法行政的轨道上有效运行。

　　着力加强行政效能建设。进一步优化投资创业环境。继续清理行政审批事项，推进部门审批职能向审批办集中，审批办向政务大厅集中，审批权限向服务窗口集中；规范各级政务服务中心及分中心运行管理，加快建设网上申报审批试点。大力推行即办制和重大项目全程领办代办制，全面推广一次性告知、一站式办理服务模式，努力为投资者和广大群众提供零距离、无障碍、低成本服务。适时推进事业单位分类改革，不断提高公益服务水平。进一步创造便民利民条件。深入推进政务公开，所有面向社会服务的政府部门和公共企事业单位，都要全面推行办事公开制度，充分保障人民群众的知情权、参与权和监督权；加强政府网站、市长公开电话、市长电子邮箱、媒体市民热线、110、120等公共服务平台建设，快速处理群众诉求，及时解决群众切身利益问题。进一步完善绩效评估体系。严格实施绩效管理调度督查考核办法，将十项攻坚等重点工作全部纳入责任推进范畴，把考核结果作为班子评定、干部升迁、绩效奖励的主要依据，消除不想作为、不会作为现象，形成以实际成果评议广大干部的激励机制。

　　着力加强勤政廉政建设。围绕发展上水平、干部下基层，深入开展“三帮双促”活动，促进机关作风的进一步转变。大兴务实重行之风，督导各级干部履职尽责，真正把精力集中到推动发展上，把心思发挥到狠抓落实上，始终保持脚踏实地、只争朝夕的精神状态和工作劲头。大兴开拓创新之风，以开放的视野，改革的魄力，积极探索改进工作、解决问题、提高效率的新思路、新举措和新办法，不断增强创新发展的素质和本领。大兴为民谋利之风，从群众最关心的事情抓起，从群众最困难的事情帮起，从群众最期望的事情做起，更大程度地解决人民群众反映强烈的突出问题。大兴勤俭廉洁之风，严格预算约束，加强财政监督，降低行政运行成本，有效促进节约型机关建设；深入落实党风廉政建设责任，加大行政执法监察力度，严厉查处违法违纪案件。

　　各位代表，吉林市正处在加快发展的关键时期。形势催人奋进，任务艰巨繁重，未来充满希望。让我们在省委省政府和市委的正确领导下，在市人大、市政协的大力支持下，更加紧密地团结起来，以昂扬向上的激情斗志、一往无前的拼搏干劲，锐意进取，奋力跨越，全面开创科学发展、富民强市的崭新局面！
